# Using Machine Learning to Identify Geographic and Socioeconomic Disparities in Dialysis Facility Outcomes Across the United States

**DOI:** 10.31486/toj.25.0040

**Published:** 2025

**Authors:** Ziad M. Ashkar, Raju Gottumukkala

**Affiliations:** ^1^ Department of Nephrology, Ochsner Lafayette General Medical Center, Lafayette, LA; ^2^ Informatics Research Institute, University of Louisiana at Lafayette, Lafayette, LA

**Keywords:** *Dialysis*, *geographic information systems*, *health status disparities*, *machine learning*, *socioeconomic factors*

## Abstract

**Background:**

Despite progress in dialysis care, the patient outcomes of mortality, hospitalization, and readmission rates remain unsatisfactory because of complex clinical, demographic, and socioeconomic interactions. For this study, we used unsupervised machine learning to identify clusters of dialysis facilities based on quality metrics and sociodemographic factors, with attention to racial and geographic disparities.

**Methods:**

We sourced facility-level data from data.cms.gov and sourced ZIP Code Tabulation Area–level sociodemographic data from the 2021 American Community Survey via the US Census Bureau application programming interface. Datasets were linked by ZIP code, standardized, and analyzed using principal component analysis and k-means clustering. We examined geographic patterns by US Census Bureau regions. Analyses were conducted in Python version 3.11.6 (Python Software Foundation) with the following libraries: pandas for data manipulation, scikit-learn for machine learning and principal component analysis, Matplotlib and Seaborn for data visualization, and GeoPandas for geographic mapping and spatial analysis.

**Results:**

Two facility clusters emerged: Cluster 0 (n=4,609) and Cluster 1 (n=2,857). Cluster 1 was characterized by poorer outcomes (higher mortality, hospitalization, readmission, anemia, catheter use, and hyperphosphatemia); lower rates of fistula use; and lower dialysis adequacy compared to Cluster 0. Cluster 1 facilities were more prevalent in regions with lower income, higher unemployment, and lower college education, and they served populations with greater proportions of Black and Hispanic residents. Geographically, Cluster 1 facilities were concentrated in the southern and western United States. Compared to Cluster 0, a larger share of Cluster 1 facilities were for-profit facilities (91.4% vs 88.5%).

**Conclusion:**

This study highlights a distinct cluster of underperforming dialysis clinics serving socioeconomically disadvantaged and racially diverse populations. Addressing these disparities requires multifaceted strategies including patient-level, institutional, and policy-level interventions.

## INTRODUCTION

Dialysis outcomes—particularly mortality, hospitalization, and readmission rates—continue to pose major clinical and public health challenges. In the United States, annual mortality rates among patients with end-stage renal disease (ESRD) on hemodialysis exceed 20%.^1^ The high rates of hospitalization and readmission in dialysis patients increase health care costs and reflect persistent gaps in care quality.^2,3^ Traditional dialysis quality metrics such as Kt/V (a ratio that assesses the adequacy of dialysis treatment where K is dialyzer clearance [how effectively dialysis removes urea from the blood], t is the dialysis session time, and V is the volume of distribution [ie, the volume of body fluid where urea is distributed]); vascular access type; and treatment adherence are associated with adverse outcomes. For instance, the use of central venous catheters and poor adherence to treatment sessions correlate with elevated risks of hospitalization and death,^4^ while hyperphosphatemia has also been linked to increased mortality.^5^ However, clinical indicators alone do not fully capture patient risk.

Machine learning is a field of artificial intelligence that uses algorithms to identify patterns in data and to make predictions or classifications based on those patterns. In biomedical applications, machine learning methods are used to analyze complex, high-dimensional datasets.^6^

Supervised machine learning is a data-driven approach that leverages labeled clinical data to develop predictive or classification models and is the predominant machine learning paradigm used in current medical research. Supervised learning encompasses a range of techniques, from traditional regression models to more complex algorithms such as random forest and support vector machine.^7^ Unlike supervised learning, which requires labeled data (eg, known diagnoses), unsupervised learning operates on unlabeled data and is particularly valuable for hypothesis generation, exploratory data analysis, and precision medicine initiatives. These unsupervised learning algorithms autonomously identify hidden patterns, groupings, or structures within the data. Common methods include clustering (eg, k-means, hierarchical clustering), dimensionality reduction (eg, principal component analysis), and feature learning.^8^

Supervised machine learning algorithms, including random forest and support vector machine, have demonstrated superior accuracy in predicting readmission and mortality among dialysis patients compared to conventional models such as linear regression.^9,10^ Machine learning models have also been used to assess the impact of quality metrics such as dialysis adequacy and anemia management.^11,12^ Unsupervised machine learning has been used to identify subpopulations of dialysis patients who have unique risk profiles for complications such as intradialytic hypotension, anemia, or infection that may not be apparent using traditional clinical criteria.^13^

Sociodemographic and regional disparities remain pivotal to understanding variation in dialysis outcomes. Golestaneh et al demonstrated that patients receiving hemodialysis in communities with a higher percentage of Black residents had a significantly higher adjusted rate of hospitalization, independent of patient comorbidities and dialysis care factors.^14^ Wilkinson et al conducted a systematic review and found that ethnic minorities, including Black and Hispanic patients, face persistent disadvantages in dialysis adequacy, access to treatment modalities, and mortality, with these disparities persisting after adjustment for clinical variables.^15^

Factors such as socioeconomic status, health literacy, and geographic access to care contribute to these disparities,^16^ underscoring the necessity of integrating social determinants of health into predictive modeling and policy planning.^17^

Substantial evidence points to geographic disparities in dialysis outcomes across the United States. For instance, a seminal analysis of United States Renal Data System data by Wetmore et al (2017) found notable regional variations in ESRD mortality and hospitalization, with the Southeast exhibiting consistently higher mortality rates despite similar clinical practices elsewhere.^18^ These disparities persisted even after adjustment for demographics and comorbidities. Staffing differences also contribute to variations in outcomes. Plantinga et al showed that dialysis facilities with the highest patient to patient care technician ratios are associated with worse patient outcomes.^19^ In an analysis of 4,800 dialysis facilities, Yoder et al reported that the average registered nurse to patient ratio was highest in the Northeast and Midwest, intermediate in the West, and lowest in the South.^20^ Access to care and regional characteristics further modulate dialysis-related risks.^21,22^

For this study, we applied cluster analysis to explore dialysis outcomes across the 50 US states, with a particular focus on identifying sociodemographic and geographic disparities.

## METHODS

Facility-level data were obtained from the Centers for Medicare & Medicaid Services (CMS) Dialysis Facility Compare (DFC) dataset, accessed via data.cms.gov, with a refresh date of January 2025. The analysis focused on the DFC_FACILITY file that includes facility-level mortality rates for the period January 1, 2020, to December 31, 2023. To ensure national representativeness, only facilities located in the 50 US states were included, resulting in a final sample of 7,488 dialysis facilities. Sociodemographic data at the ZIP Code Tabulation Area level were retrieved from the 2021 American Community Survey 5-year estimates via the US Census Bureau application programming interface. These data were linked to dialysis facility ZIP codes using a left join, achieving a 99.7% match rate (n=7,466 matched facilities).

Linked matched data (n=7,466 facilities) preprocessing was then conducted to ensure compatibility with machine learning workflows. Steps included (1) missing data handling–numerical columns with missing values were imputed using the mean of each respective feature; (2) feature scaling–numerical features were standardized to have zero mean and unit variance to ensure equal contribution across variables; and (3) dimensionality reduction–a total of 35 numerical features were included in a principal component analysis to reduce dimensionality while preserving variance. The number of components retained was determined by setting a 95% cumulative explained variance threshold. The resulting principal component analysis transformation retained 29 principal components, reflecting the dataset's high dimensionality and complexity. Principal component 1 (PC1) is the linear combination that captures the largest share of the variance in the dataset and is dominated by dialysis quality. Principal component 2 (PC2) captures the next share of variance and is dominated by demographics.

The principal component analysis–transformed data were subjected to k-means clustering to uncover natural groupings among facilities. The optimal number of clusters was determined using the silhouette score, with the number yielding the highest score selected as optimal. Final clustering was performed using k-means with this optimal cluster count.

Geographic distribution was analyzed across US states and census regions. Statistical comparisons between clusters were conducted using one-way analysis of variance for continuous variables and chi-square tests for categorical variables. All data processing, analysis, and visualization were conducted using Python version 3.11.6 (Python Software Foundation) with the following libraries: pandas for data manipulation, scikit-learn for machine learning and principal component analysis, Matplotlib and Seaborn for data visualization, and GeoPandas for geographic mapping and spatial analysis.

## RESULTS

### Cluster Analysis of Dialysis Facilities

**Figure 1.**
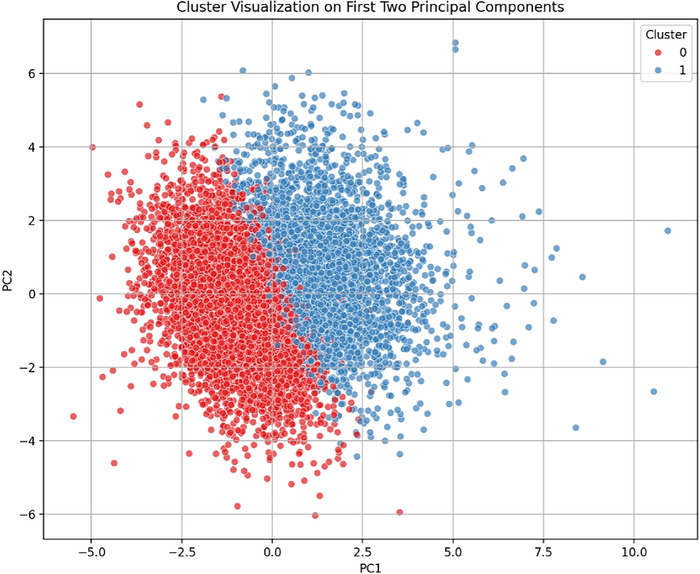
Cluster distribution of dialysis facilities using k-means clustering. Principal component 1 (PC1) is the linear combination that captures the largest share of the variance in the dataset and is dominated by dialysis quality. Principal component 2 (PC2) captures the next share of variance and is dominated by demographics.

Using principal component analysis and k-means clustering, 2 distinct groups of dialysis facilities were identified (Calinski-Harabasz score=505.83). Cluster 0 comprised 4,609 facilities (61.7%), and Cluster 1 comprised 2,857 facilities (38.3%). Figure 1 shows the cluster distribution. As discussed in the Methods section, PC1 is dominated by dialysis quality, and PC2 is dominated by demographics. Clear cluster separation is noted, supporting the validity of further analysis.

Table 1 provides a summary of dialysis facility data, with variables categorized as categorical and continuous. The dataset includes facility characteristics, services offered, patient demographics, and key clinical performance metrics.

**Table 1.** Summary of Dialysis Facility Data, n=7,466

Data	Value
**Categorical Variables—Dialysis Facility Characteristics** ^a^	
Business type	
For-profit	6,692 (89.6)
Nonprofit	774 (10.4)
Chain owned	
Yes	6,739 (90.3)
No	727 (9.7)
Offers late shift	
Yes	1,163 (15.6)
No	6,303 (84.4)
Offers in-center hemodialysis	
Yes	6,958 (93.2)
No	508 (6.8)
Offers peritoneal dialysis	
Yes	4,046 (54.2)
No	3,420 (45.8)
Offers home hemodialysis training	
Yes	2,348 (31.4)
No	5,118 (68.6)
**Continuous Variables—Dialysis Facility Characteristics** ^b^	
Number of dialysis stations	17.5 ± 8.6
Mortality rate (deaths/100 patient-years)	22.2 ± 5.7
Hospitalization rate (hospitalizations/100 patient-years)	141.4 ± 38.9
Readmission rate	26.3 ± 8.0
Transfusion rate	31.0 ± 22.1
Patients waitlisted for transplant	16.1 ± 9.9
Standardized first kidney transplant waitlist ratio	0.99 ± 0.81
Standardized emergency department visits ratio	1.0 ± 0.4
Standardized emergency department visits within 30 days of hospital discharge ratio	1.1 ± 0.5
Standardized modality switch ratio^c^	1.1 ± 1.0
Standard infection ratio	0.3 ± 0.4
Fistula rate	58.5 ± 11.7
**Continuous Variables—Dialysis Facility Patient Demographics** ^b^	
Age >65 years	16.3 ± 6.1
Male^d^	49.0 ± 3.2
Race/ethnicity^d^	
White non-Hispanic	54.5 ± 27.2
Black non-Hispanic	17.2 ± 21.1
Asian	5.2 ± 8.5
Two or more races	3.1 ± 2.2
Hispanic/Latino	18.6 ± 21.3
Bachelor's degree or higher	30.1 ± 10.0
Household income, $, mean median	67,501.1 ± 26,252.0
Unemployed	2.9 ± 1.3
**Continuous Variables—Dialysis Facility Patient Clinical Characteristics** ^b^	
Kt/V ≥1.2	97.0 ± 6.7
Serum calcium >10.2 mg/dL	1.8 ± 5.9
Hemoglobin	
<10 g/dL	20.4 ± 12.9
>12 g/dL	0.56 ± 2.1
Serum phosphorus	
<3.5 mg/dL	7.4 ± 3.0
3.6 to 4.5 mg/dL	22.8 ± 5.1
4.6 to 5.5 mg/dL	28.0 ± 4.8
5.6 to 7.0 mg/dL	24.7 ± 5.0
>7.0 mg/dL	17.1 ± 6.0
Long-term catheter in use	18.0 ± 9.3

^a^Categorical variables data are reported as n (%).

^b^Continuous variables data are reported as percentage means ± SDs, except number of dialysis stations that is reported as mean ± SD and household income that is reported as mean median.

^c^The standardized modality switch ratio is the ratio of observed to expected switches from home dialysis (either peritoneal dialysis or home hemodialysis) to in-center hemodialysis.

^d^Sex and race were considered continuous variables for the purpose of statistical modeling.

Table 2 shows dialysis facility characteristics by cluster for the continuous variables. Compared to Cluster 0 facilities, Cluster 1 facilities had higher mortality, hospitalization, and readmission rates and worse quality metrics: worse clearance (percent of patients with Kt/V >1.2), lower fistula use, and worse anemia (patients with hemoglobin <10 g/dL) and hyperphosphatemia (patients with serum phosphorus >7 mg/dL) management. Cluster 1 facilities also had a lower percentage of patients waitlisted for transplant. Moreover, higher percentages of Black and Hispanic patients were treated at Cluster 1 facilities, and overall, the patients at Cluster 1 facilities had lower household incomes, a lower percentage of college educations, and higher unemployment rates compared to patients at Cluster 0 facilities.

**Table 2.** Dialysis Facility Characteristics by Cluster, Continuous Variables

Continuous Variables	Cluster 0, n=4,609	Cluster 1, n=2,857	*P* Value
**Dialysis Facility Characteristics**			
Number of dialysis stations	15.74 ± 7.64	20.36 ± 9.27	<0.0001
Mortality rate (deaths/100 patient-years)	21.89 ± 5.60	22.57 ± 5.82	<0.0001
Hospitalization rate (hospitalizations/100 patient-years)	138.19 ± 37.87	146.44 ± 39.85	<0.0001
Readmission rate	25.47 ± 8.21	27.59 ± 7.45	<0.0001
Transfusion rate	30.58 ± 21.90	31.76 ± 22.34	0.066
Patients waitlisted for transplant	16.96 ± 10.38	14.68 ± 8.95	<0.0001
Standardized first kidney transplant waitlist ratio	1.19 ± 0.86	0.75 ± 0.64	<0.0001
Standardized emergency department visits ratio	1.01 ± 0.43	0.99 ± 0.42	0.265
Standardized emergency department visits within 30 days of hospital discharge ratio	1.07 ± 0.51	1.03 ± 0.48	0.003
Standardized modality switch ratio^a^	1.34 ± 1.11	0.72 ± 0.62	<0.0001
Standard infection ratio	0.35 ± 0.44	0.27 ± 0.33	<0.0001
Fistula rate	59.67 ± 11.0	56.57 ± 12.48	<0.0001
**Dialysis Facility Patient Demographics**			
Age >65 years	18.08 ± 6.58	13.51 ± 3.95	<0.0001
Male^b^	49.24 ± 2.75	48.74 ± 3.75	<0.0001
Race/ethnicity^b^			
White non-Hispanic	71.20 ± 16.73	27.52 ± 17.55	<0.0001
Black non-Hispanic	8.79 ± 9.86	30.68 ± 26.62	<0.0001
Asian	5.10 ± 8.21	5.34 ± 8.84	0.246
Two or more races	3.41 ± 2.30	2.57 ± 1.82	<0.0001
Hispanic/Latino	10.41 ± 9.61	31.82 ± 27.36	<0.0001
Bachelor's degree or higher	33.54 ± 9.51	24.54 ± 7.99	<0.0001
Household income, $, mean median	74,277.45 ± 27,063.30	56,528.85 ± 20,566.79	<0.0001
Unemployed	2.42 ± 0.93	3.74 ± 1.36	<0.0001
**Dialysis Facility Patient Clinical Characteristics**			
Kt/V ≥1.2	97.68 ± 3.97	95.88 ± 9.37	<0.0001
Serum calcium >10.2 mg/dL	1.31 ± 2.72	2.63 ± 7.85	<0.0001
Hemoglobin			
<10 g/dL	19.50 ± 12.38	21.69 ± 13.49	<0.0001
>12 g/dL	0.63 ± 2.26	0.44 ± 1.87	0.001
Serum phosphorus			
<3.5 mg/dL	6.97 ± 2.64	8.07 ± 3.42	<0.0001
3.6 to 4.5 mg/dL	22.88 ± 5.11	22.68 ± 5.10	0.1
4.6 to 5.5 mg/dL	28.38 ± 4.81	27.49 ± 4.77	<0.0001
5.6 to 7.0 mg/dL	24.83 ± 4.97	24.41 ± 4.96	0.0004
>7.0 mg/dL	16.94 ± 5.87	17.36 ± 6.17	0.005
Long-term catheter in use	17.63 ± 8.73	18.63 ± 10.01	<0.0001

Note: Data are reported as percentage means ± SD, except number of dialysis stations that is reported as mean ± SD and household income that is reported as mean median.

^a^The standardized modality switch ratio is the ratio of observed to expected switches from home dialysis (either peritoneal dialysis or home hemodialysis) to in-center hemodialysis.

^b^Sex and race were considered continuous variables for the purpose of statistical modeling.

Table 3 shows dialysis facility characteristics by cluster for the categorical variables. Compared to Cluster 0, a higher percentage of Cluster 1 facilities—the cluster with worse mortality, hospitalization, and readmission outcomes—were for-profit and offered late shifts (late afternoon or early evening timing for dialysis). In addition, lower percentages of Cluster 1 facilities offered peritoneal dialysis and home hemodialysis training compared to Cluster 0 facilities.

**Table 3.** Dialysis Facility Characteristics by Cluster, Categorical Variables

Categorical Variables	Cluster 0, n=4,609	Cluster 1, n=2,857	*P* Value
Business type			<0.0001
For-profit	4,080 (88.5)	2,612 (91.4)	
Nonprofit	529 (11.5)	245 (8.6)	
Chain owned			<0.0001
Yes	4,218 (91.5)	2,521 (88.2)	
No	391 (8.5)	336 (11.8)	
Offers late shift			0.034
Yes	685 (14.9)	478 (16.7)	
No	3,924 (85.1)	2,379 (83.3)	
Offers in-center hemodialysis			<0.0001
Yes	4,237 (91.9)	2,721 (95.2)	
No	372 (8.1)	136 (4.8)	
Offers peritoneal dialysis			<0.0001
Yes	2,696 (58.5)	1,350 (47.3)	
No	1,913 (41.5)	1,507 (52.7)	
Offers home hemodialysis training			<0.0001
Yes	1,641 (35.6)	707 (24.7)	
No	2,968 (64.4)	2,150 (75.3)	

Note: Data are reported as n (%).

**Figure 2.**
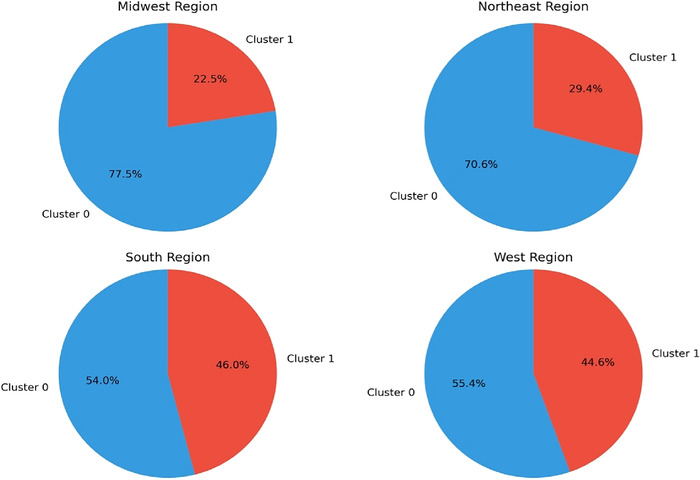
Cluster 0 and Cluster 1 dialysis facility distribution by US Census Bureau region.

To determine cluster distribution by region, the 50 US states were mapped by US Census Bureau region.^23^ Figure 2 shows the cluster distribution by region. The Midwest and Northeast regions have the highest proportion of Cluster 0 facilities (77.5% and 70.6%, respectively), while the South and West regions have the highest proportion of Cluster 1 facilities (46.0% and 44.6%, respectively).

**Figure 3.**
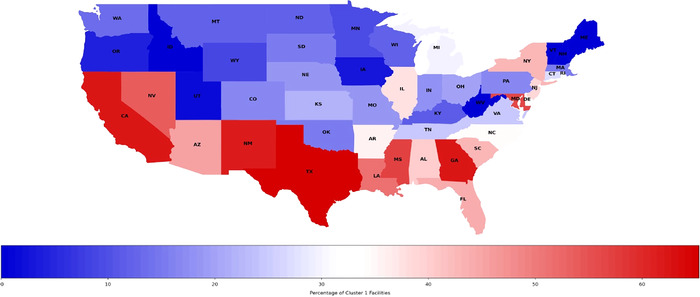
Percentages of Cluster 1 dialysis facilities by state. The 9 states with the highest percentages of Cluster 1 facilities are Texas (65%), California (62%), Georgia (62%), New Mexico (61%), Mississippi (57%), Nevada (54%), Louisiana (51%), Arizona (45%), and Florida (33%).

Figure 3 shows Cluster 1 facility distribution in the 48 contiguous states. States with high percentages of Cluster 1 facilities are principally located in the South and West. The 9 states with the highest percentages of Cluster 1 facilities are Texas (65%), California (62%), Georgia (62%), New Mexico (61%), Mississippi (57%), Nevada (54%), Louisiana (51%), Arizona (45%), and Florida (33%).

Table 4 shows economic indicators by US Census Bureau region. The lowest median household income and lowest rate of college education are in the South.

**Table 4.** Economic Indicators by US Census Bureau Region Where Dialysis Facilities Are Located

US Census Bureau Region	Median Household Income, $	Unemployment Rate, %	College Education Rate, %
Northeast	77,231.98	3.16	32.57
Midwest	64,948.23	2.81**	30.87
South	61,877.57*	2.85	28.67***
West	76,971.56	3.09	30.83

*P<0.0001 by analysis of variance for median household income.

**P<0.0001 by analysis of variance for unemployment rate.

***P<0.0001 by analysis of variance for college education rate.

**Figure 4.**
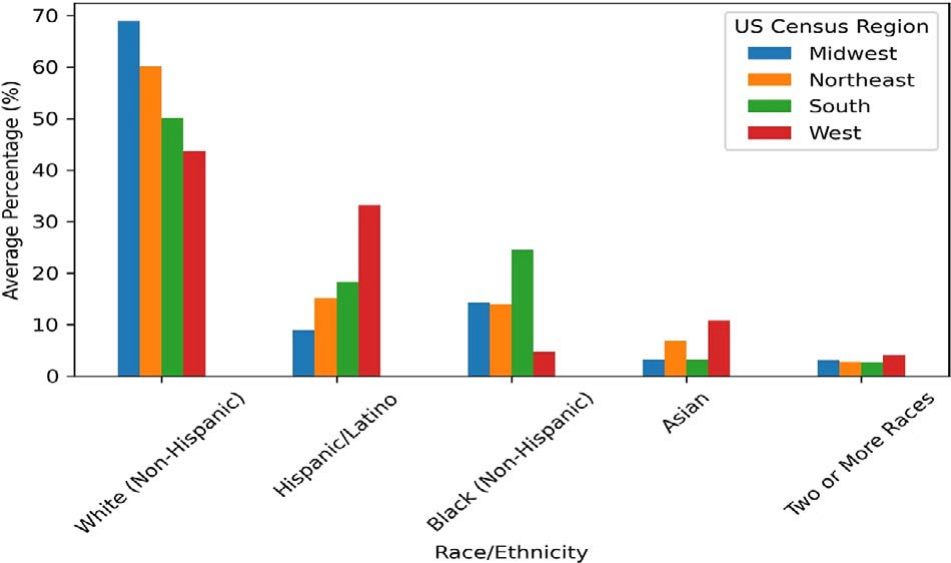
Racial/ethnic composition comparison across US Census Bureau regions.

Figure 4 shows the racial/ethnic distribution by US Census Bureau region of the population where the dialysis clinics are located. The highest percentage of Blacks (24.5%) is in the South, and the highest percentages of Asians (10.8%) and Hispanics/Latinos (33.2%) are in the West. The highest percentages of Whites are in the Midwest (69.0%) and Northeast (60.2%).

Table 5 shows dialysis facility quality indicators (outcomes) by US Census Bureau region. The South has the highest mortality rate, lowest fistula rate, highest anemia rate, highest hyperphosphatemia rate, highest percentage of for-profit facilities, and the lowest percentage of facilities offering home hemodialysis training. The West has the second highest mortality rate, the second highest hyperphosphatemia rate, and second lowest percentage of facilities offering home hemodialysis training. Noticeably, the West has the highest percentage of facilities offering late shifts and highest number of dialysis stations.

**Table 5.** Dialysis Facility Quality Indicators by US Census Bureau Region

	US Census Bureau Region				
Quality Indicator	Northeast	Midwest	South	West	*P* Value
**Continuous Variables^a^**					
Number of dialysis stations	18.04 ± 7.99	15.03 ± 8.10	17.90 ± 8.49	19.07 ± 9.26	<0.0001
Mortality rate (deaths/100 patient-years)	19.67 ± 5.15	20.82 ± 5.39	23.55 ± 5.74	22.14 ± 5.32	<0.0001
Hospitalization rate (hospitalizations/100 patient-years)	145.97 ± 36.35	142.80 ± 39.57	144.88 ± 40.39	127.73 ± 32.41	<0.0001
Readmission rate	27.48 ± 7.61	25.78 ± 8.20	26.51 ± 7.92	25.46 ± 8.08	<0.0001
Patients waitlisted for transplant	23.41 ± 10.91	14.12 ± 8.54	13.92 ± 8.05	18.36 ± 11.61	<0.0001
Fistula rate	61.04 ± 11.84	56.95 ± 11.66	56.38 ± 11.54	63.47 ± 10.05	<0.0001
Kt/V ≥1.2	96.47 ± 8.27	97.48 ± 4.42	96.79 ± 7.47	97.22 ± 5.40	0.00058
Hemoglobin <10 g/dL	21.13 ± 14.60	20.57 ± 13.33	21.42 ± 12.69	17.24 ± 10.83	<0.0001
Serum calcium >10.2 mg/dL	2.37 ± 7.87	1.29 ± 2.81	1.92 ± 6.53	1.79 ± 5.34	<0.0001
Serum phosphorus >7.0 mg/dL	14.44 ± 4.22	16.67 ± 5.96	17.87 ± 6.04	17.65 ± 5.86	<0.0001
Long-term catheter in use	18.52 ± 10.11	19.81 ± 9.02	17.29 ± 9.12	17.38 ± 9.06	<0.0001
**Categorical Variables** ^b^					
For-profit	85.65	88.36	93.15	85.47	<0.0001
Offers late shift	23.96	12.37	10.73	24.94	<0.0001
Offers peritoneal dialysis	54.76	53.94	53.39	56.00	0.41
Offers home hemodialysis training	34.55	34.68	29.52	30.12	0.00002

^a^Continuous variables data are reported as percentage means ± SD, except number of dialysis stations that is reported as mean ± SD.

^b^Categorical variables data are reported as percentages.

## DISCUSSION

For this study, we used unsupervised machine learning to analyze data from CMS dialysis facilities and the US Census Bureau. Facilities in Cluster 1 exhibited worse clinical outcomes, including higher mortality, hospitalization, and readmission rates than facilities in Cluster 0. These outcomes were associated with lower quality metrics, such as suboptimal metrics in dialysis clearance (Kt/V ≥1.2), mineral bone disease (serum phosphorus >7.0 mg/dL), anemia (hemoglobin <10 g/dL), and vascular access (fistula rate and long-term catheter use).

These findings are consistent with prior research. For instance, anemia, particularly when severe, is a known predictor of mortality and hospitalization during maintenance dialysis.^24,25^ Lacson et al showed that dialysis facilities with better performance in vascular access use, anemia management, and dialysis clearance demonstrated lower mortality and hospitalization rates.^26^ Suboptimal control of mineral metabolism—such as abnormally high phosphorus, calcium, or parathyroid hormone levels—has also been linked to increased cardiovascular mortality and hospitalizations.^27^ In our study, we found higher percentages of hyperphosphatemia (phosphorus >7 mg/dL) and hypercalcemia (calcium >10.2 mg/dL) in Cluster 1 compared to Cluster 0; however, noticeably, Cluster 1 had a higher percentage of hypophosphatemia (phosphorus <3.5 mg/dL) than Cluster 0. Hypophosphatemia has been linked to increased mortality in dialysis patients, and has also been associated with hypoalbuminemia, reflecting poor nutritional and functional status.^28,29^

Cluster 1 facilities had a lower percentage of patients aged 65 years or older than Cluster 0 facilities. Villar et al showed that older patients with ESRD experienced less excess mortality than younger patients with ESRD.^30^ Kucirka et al compared mortality among races and showed that Black patients on dialysis had a lower risk of death compared to White patients, but this survival advantage only applied to older adults (>50 years).^31^ Of note, the Cluster 1 facilities in our study had a higher percentage of Black patients than the Cluster 0 facilities.

Compared to Cluster 0 facilities, Cluster 1 facilities were also notable for a lower percentage of patients on the transplant waitlist, a factor known to be associated with increased mortality in dialysis.^32^ Compared to Cluster 0 facilities, Cluster 1 facilities also had a higher proportion of for-profit clinics, a finding that has been associated with adverse outcomes, including higher rates of mortality and hospitalization.^33,34^ Contributing factors include staffing, resource allocation, financial pressures, and the likelihood of referral for kidney transplant.^35^

In our study, facilities in Cluster 1 had a larger average number of dialysis stations and a greater likelihood of offering late shifts. Studies have shown an increased mortality risk in the smallest facilities (≤15 stations) but no demonstrated difference in mortality or quality outcomes in facilities with 16 to 20 stations vs facilities with >20 stations.^36,37^

Peritoneal dialysis and home hemodialysis have been associated with improved patient outcomes, including reduced mortality and hospitalization.^38-40^ In their systematic review, Walker et al found that patients on home hemodialysis reported satisfaction with staff expertise and educational support.^41^ In our study, a lower percentage of Cluster 1 facilities offered peritoneal dialysis and home hemodialysis training compared to Cluster 0 facilities.

Mapping by US Census Bureau regions revealed that Cluster 1 facilities were principally concentrated in the South and West. State-level analysis further confirmed that states in the South and West had the highest proportions of Cluster 1 facilities. Some of our findings align with previous data that report higher dialysis mortality and hospitalization rates in the South compared to other US regions,^42^ although mortality in the West has been reported to be lower than in the South and lower to comparable compared to mortality in the Midwest and Northeast.^43^ To further understand these potential conflicting differences—given that we showed higher mortality in the West—we looked at the sociodemographic analysis of the US Census Bureau regions. These data showed that the South had the lowest median household income and college education rate among the 4 regions. The South also had the highest proportion of Blacks in the covered ZIP codes of the dialysis service areas, while the West had the highest proportion of Hispanics in the covered ZIP codes of the dialysis service areas. As noted previously, prior studies have demonstrated strong associations between worse health outcomes and socioeconomic disadvantage and racial minority status.^14,15^ When we compared regional quality metrics and other dialysis facility characteristics by US Census Bureau region, we found that the South had the highest mortality rate, lowest fistula rate, highest anemia rate, highest hyperphosphatemia rate, highest percent of for-profit facilities, and lowest percent of facilities offering home hemodialysis training. In comparison, the West had the second highest mortality rate, second highest hyperphosphatemia rate, and the second lowest percentage of clinics offering home hemodialysis training. Together, these factors—clinical performance, facility characteristics, and sociodemographic context—provide a reasonable explanation for the geographic concentration of Cluster 1 facilities in the South and West.

Based on our study findings, there is a great need for multidisciplinary interventions focused on care access, resource allocation, and dialysis quality metrics to yield improvements in dialysis patient outcomes. Further research should incorporate patient-level comorbidity data, staffing models, and regional policy analysis to further refine our understanding of mortality and quality disparities in ESRD care. Ultimately, addressing these inequities will require targeted clinical, operational, and policy-level reforms to ensure equitable access to high-quality dialysis care across all communities.

This study has several limitations. First, as an unsupervised machine learning study, clustering techniques rely on input variables, and some outcome predictors may not have been captured. Second, our analysis is based on facility-level data that may not fully reflect individual patient characteristics or comorbidities, such as nutritional status and diabetes. Third, confounding factors such as local health care policies, insurance coverage, and referral practices were not directly analyzed. Strengths of the study are its national scope and integration of facility quality metrics with community-level sociodemographic data. The unsupervised machine learning approach allowed for data-driven discovery of latent facility groupings not constrained by a priori assumptions.

## CONCLUSION

This study used unsupervised machine learning to identify clusters of dialysis facilities with distinct quality profiles, uncovering significant disparities in mortality, hospitalization, and readmission rates. Facilities in Cluster 1—representing nearly 40% of the national sample—were disproportionately located in socioeconomically disadvantaged areas—predominantly in the South and West regions of the United States—and served racially diverse populations. These facilities exhibited poorer performance across key quality indicators, including vascular access, dialysis adequacy, anemia management, hyperphosphatemia, and transplant waitlisting compared to the Cluster 0 facilities. The findings underscore a pressing need for multifaceted interventions including allocating resources to high-risk regions, implementing programs focused on improving quality processes (such as vascular access, mineral bone disease control, and anemia management), expanding access to home dialysis training, and enhancing transplant waitlist processes in underserved areas. Addressing these factors could potentially improve patient outcomes. Our study highlights the utility of machine learning approaches in uncovering systemic patterns in health care delivery. By integrating facility-level quality metrics with sociodemographic context, this study offers a novel framework for identifying geographic and structural disparities in dialysis care.

## Notes

*This article meets the Accreditation Council for Graduate Medical Education and the American Board of Medical Specialties Maintenance of Certification competencies for Patient Care and Medical Knowledge.*
